# Broader Impacts for Ecologists: Biological Soil Crust as a Model System for Education

**DOI:** 10.3389/fmicb.2020.577922

**Published:** 2021-01-05

**Authors:** Akasha M. Faist, Anita J. Antoninka, Nichole N. Barger, Matthew A. Bowker, V. Bala Chaudhary, Caroline A. Havrilla, Elisabeth Huber-Sannwald, Sasha C. Reed, Bettina Weber

**Affiliations:** ^1^Department of Animal and Range Sciences, New Mexico State University, Las Cruces, NM, United States; ^2^School of Forestry, Northern Arizona University, Flagstaff, AZ, United States; ^3^Department of Ecology and Evolutionary Biology, University of Colorado, Boulder, CO, United States; ^4^Department of Environmental Science and Studies, DePaul University, Chicago, IL, United States; ^5^US Geological Survey, Southwest Biological Science Center, Flagstaff, AZ, United States; ^6^Department of Biological Sciences, Northern Arizona University, Flagstaff, AZ, United States; ^7^División de Ciencias Ambientales, Instituto Potosino de Investigación Científica y Tecnológica, San Luis Potosí, Mexico; ^8^US Geological Survey, Southwest Biological Science Center, Moab, UT, United States; ^9^Department of Biology, University of Graz, Graz, Austria; ^10^Multiphase Chemistry Department, Max Planck Institute for Chemistry, Mainz, Germany

**Keywords:** biocrust, ecology, evolution, scale, patterns and processes, succession, classroom

## Abstract

Biological soil crusts (biocrusts) are a complex community of algae, cyanobacteria, lichens, bryophytes, and assorted bacteria, fungi, archaea, and bacteriophages that colonize the soil surface. Biocrusts are particularly common in drylands and are found in arid and semiarid ecosystems worldwide. While diminutive in size, biocrusts often cover large terrestrial areas, provide numerous ecosystem benefits, enhance biodiversity, and are found in multiple configurations and assemblages across different climate and disturbance regimes. Biocrusts have been a focus of many ecologists, especially those working in semiarid and arid lands, as biocrusts are foundational community members, play fundamental roles in ecosystem processes, and offer rare opportunities to study biological interactions at small and large spatial scales. Due to these same characteristics, biocrusts have the potential to serve as an excellent teaching tool. The purpose of this paper is to demonstrate the utility of biocrust communities as a model system in science education. Functioning as portable, dynamic mini ecosystems, biocrusts can be used to teach about organisms, biodiversity, biotic interactions, abiotic controls, ecosystem processes, and even global change, and can be easy to use in nearly every classroom setup. For example, education principles, such as evolution and adaptation to stress, or structure and function (patterns and processes) can be applied by bringing biocrusts into the classroom as a teaching tool. In addition, discussing the utility of biocrusts in the classroom – including theory, hypothesis testing, experimentation, and hands-on learning – this document also provides tips and resources for developing education tools and activities geared toward impactful learning.

## Introduction

### What Are Biocrusts?

**Figure 1:**
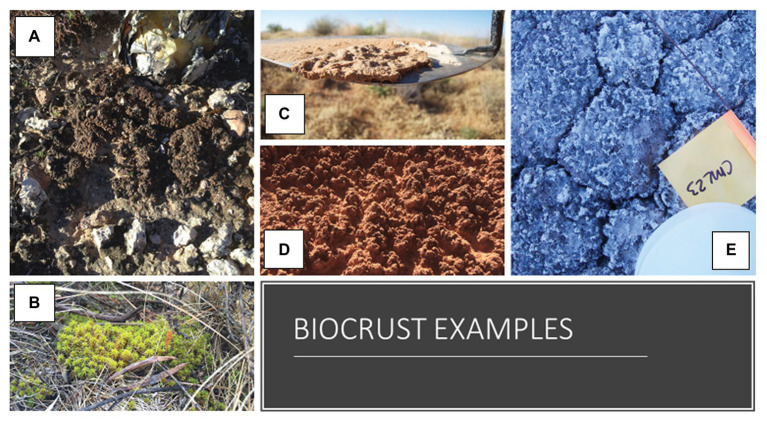
Photographs of biological soil crusts in the field to highlight their variability in color and shape, as well as their ability to hold the soil surface in place. **(A)** Top left is a lichen dominated biocrust, **(B)** bottom left is a moss dominated biocrust, and **(C–E)** in the middle and on the right are examples of cyanobacteria dominated biocrusts.

Biological soil crusts (biocrusts; examples provided in Figure 1) are defined as a photosynthetic surface soil community living in and binding together the top millimeters of soil (Belnap and Lange, 2001; Weber et al., 2016). Within this community, the common major players are cyanobacteria, algae, bryophytes, and lichens, and these primary producers provide habitat and food for a diverse soil food web, including bacteria, fungi, diatoms, protozoa, nematodes, and microarthropods (Belnap and Lange, 2001; Darby and Neher, 2016). Biocrusts are widespread and common across the globe, covering about 12% of Earth’s terrestrial surface (Rodriguez-Caballero et al., 2018a). They are present in all ecosystems where light reaches the soil surface and thrive in places where vascular plants are less dominant, which can include harsh environments, like polar deserts, high alpine zones, hyper-arid deserts, gypsiferous, or saline soils, as well as arid and semiarid regions. Biocrusts also are commonly a successional step in areas where disturbance has exposed the soil surface to light and thus you can see biocrusts in wetter ecosystems on soil that has been exposed (e.g., from road cuts, fires, or at the forefront of receding glaciers). Biocrusts contain all domains of life, supporting a complex soil food web, and performing all vital ecosystem functions, including primary production, nitrogen fixation, aggregation, carbon storage, and stabilization of soils and regulation ecosystem hydrology (Maestre et al., 2011; Darrouzet-Nardi et al., 2015; Barger et al., 2016; Weber et al., 2016; Chamizo et al., 2017; Faist et al., 2017; Eldridge et al., 2020). While biocrusts are vitally important, they are also vulnerable to changing climate and land use disturbances (Ferrenberg et al., 2015; Rodriguez-Caballero et al., 2018a). This mix of diversity of taxa, growth forms, function, and distribution, coupled with their responsiveness to our changing world (Reed et al., 2016), make biocrusts an ideal hands-on learning system for a variety of topics.

### Biocrusts as a Model System for Teaching

For research, a model system is defined as a system that “displays a general process or property of interest, in a way that makes it understandable” (Vitousek, 2002). The utility of biocrusts as a model system in scientific studies has been clearly demonstrated (Bowker et al., 2014; Maestre et al., 2016) and provides a path forward for investigating the use of biocrusts as a model system in education. Biocrust research has increased exponentially in the last two decades, yet English language publications describing the use of biocrusts as a teaching tool have not followed. Nevertheless, educators have clearly stated that biocrusts would be of interest to use in their classroom and can act as an engaging tool to develop learning of core concepts. This interest and availability, whether field collected or grown in the classroom, coupled with biocrusts’ portable size, communities that range from simple to complex, wide span of bright colors and shapes (Figure 1), and visually dramatic transformations after being wetted from desiccated dormancy, highlight the potential experiential learning power of using biocrusts in the classroom. Further, although biocrusts are small in stature, they play extremely important roles in a wide range of ecosystems and thus offer a logistically feasible option for hands-on student experience with a foundational biological community. The range in complexity of the different topic areas means that distinct aspects of biocrust biology and ecology could better suit a range of learning phases. For example, topics related to understanding biodiversity and examining ecosystem function can be used for university level students, while questions of taxonomy and morphology lessons could fit remarkably well into the new United States K-12 standards for Science, Technology, Engineering, and Mathematics (STEM) education called “three-dimensional learning.”^1^ This three-dimensional learning uses cross-cutting concepts that have applicability across all fields of science and provide real-world research practices to teach core concepts in Physical Science, Life Science, Earth and Space Science, and Engineering. Similar policies and standards can be met for similar age groups across Europe (European Commission/EACEA/Eurydice, 2019) and throughout other countries.

**Figure 2:**
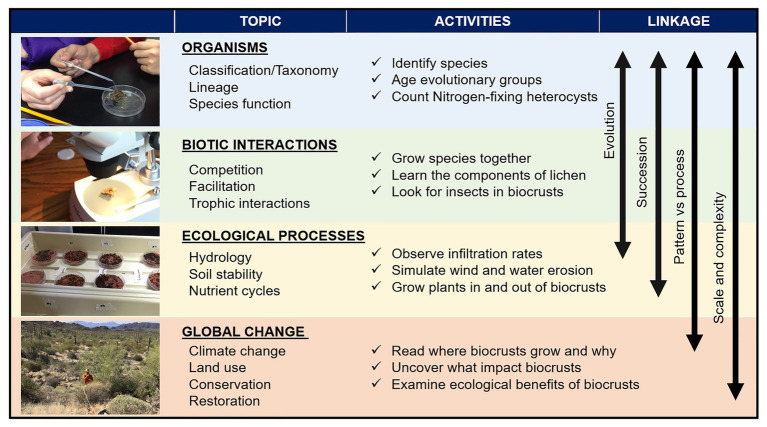
Main study fields to be investigated in the classroom with subtopics, potential activities linked to them, and the underlying concepts linking the different topics. The photos illustrate from top to bottom a biocrust moss and giving a simple water addition to demonstrate how quickly biocrusts can hydrate and change form. Below is a microscope exercise where students identify the taxa within a small piece of biocrust with multiple species living in close proximity. Next is an experiment looking at how biocrust respond to different drought conditions, and finally a survey in the field to identify biocrust diversity, abundance, and impacts from land use.

To use field collected biocrust samples in the classroom, local regulations must be met and guidelines for effective collection and cultivation of biocrust can be found in an ongoing biocrust restoration manual^2^ and adapted for educational purposes specific to each classroom. Cultivating biocrust as a classroom project can be included into the curriculum with a terrarium of different collected components of biocrust communities (e.g., cyanobacterial filaments and moss spores) to then build on the concepts and activities discussed here. We provide suggestions for using biocrusts; however, many parallel learning tools apply using virtual efforts such as photographs or videos of filamentous cyanobacteria (e.g., https://3dmoss.berkeley.edu/). As a model system, there are numerous creative ways to use biocrusts in the classroom and in the field. We link four uses specifically positioned to highlight ecological and evolutionary principles taught across science-based curricula with associated activities in each (Figure 2).

## Potential Concepts Using Biocrusts

### Evolution

In plant and other evolution courses, the curriculum often starts with the cellular organisms that evolved earlier (e.g., cyanobacteria, then eukaryotic algae), and then works through to the spore producing non-vascular plants (e.g., mosses), before focusing on vascular plants. Those earlier life forms, cyanobacteria and algae are major components of biocrusts and can be easily identified through a hand lens or microscope, growing directly adjacent to later evolved organisms such as mosses and liverworts. Instructors can guide students through direct objectives to uncover evolutionary processes at the organismal level all within one petri dish (Figure 2). Students can learn about how studies using genetics can help use understand the multifaceted unseen microbial communities that are aiding in enhancing biocrust biodviersity (Liu et al., 2017; Van Goethem et al., 2019). Students can also use a hand lens or microscope to see the structure of an individual organism in order to learn taxonomy, evolutionary adaptations, and timelines in Earth’s history (e.g., cyanobacteria ≥2 billion years prior to current time vs. moss ~450 million years prior). The communities can be linked to how evolution and ecology are intertwined and, while the different groups originated at such different times in history, show that they are still living together in similar space and time under current conditions. Biocrusts can also be incorporated into lesson plans about early colonization of land (Beraldi-Campesi and Retallack, 2016), as well as species and organismal relationships and interactions with each other (for example, facilitation and competition), which are central to ecology and evolutionary biology.

### Succession

Succession refers to predictable changes in species composition over time as an ecosystem develops (primary succession; e.g., after a glacier recedes or lava cools) or recovers from a disturbance (secondary succession; e.g., after a forest fire or big flood). Biocrusts and their associated species demonstrate a general successional trajectory and predictable community assembly in both primary and secondary successional settings (Weber et al., 2016). As they quickly colonize soils, filamentous cyanobacteria can stabilize and fertilize the soil surface to then allow subsequent successional species, such as lichens and mosses, to colonize the soil surface. Observation of biocrust successional stages, whether in the classroom terrarium or in photographs, can be used to introduce actionable objectives relevant to all ecosystems at small to large scales, such as identifying similarities and differences in primary vs. secondary succession, and juxtaposing Clementsian climax models of succession vs. alternative ecosystem states (Bowker, 2007). Biocrusts are a great system to highlight specific organisms and their influence on the environment and thereby to underscore how changes to these communities can feedback to ecosystem processes and function through time.

### Patterns and Processes

The ecological services and functions of biocrusts have been studied across systems (e.g., Belnap and Lange, 2001; Maestre et al., 2011; Weber et al., 2016; Faist et al., 2017; Rodriguez-Caballero et al., 2018b). In addition to identifying patterns of biocrust community assemblage, building the objective of understanding ecological processes can be gleaned through using biocrusts as engineering systems to test specific hypotheses and simulate system functions in the classroom. The random vs. organized patterns, or what we can see from a picture or directly through a microscope, can be used for students to document what they see at the organism level (Figure 2). In addition to documenting the magnified organisms and the patterns and shapes they create inside a petri dish, students can also learn about physiological and ecological processes.

The green color in the cells of biocrusts is due to photosynthetic pigments, performing the process of photosynthesis that is the foundation of most ecosystem functions and of the larger food web. Likewise, students can observe and quantify cyanobacterial heterocysts that are responsible for nitrogen fixation, which allows some organisms (those that can fix N_2_) to access the enormous pool of nitrogen in the atmosphere that is unavailable to most organisms. These nitrogen-fixers then release this nitrogen (for example, when they die and decompose) and, because all living things need nitrogen, this process – nitrogen fixation – sustains life on Earth. All this together and other unmentioned examples allow students to see evidence of both the small-scale patterns (i.e., what they can see from a photo or under a microscope) and processes of biocrusts and of the larger ecosystem level processes they control. From here, the function of a single organism can be connected with the provision of nutrients across a landscape, which again demonstrates concrete links between pattern and process.

Another ecosystem process well-illustrated by biocrusts is soil erosion and its control. Filamentous cyanobacteria, mosses, and lichens all can bind together the soil surface and greatly reduce erosion (Chamizo et al., 2017; Kheirfam et al., 2017). A tangible demonstration of the erosion concept can be achieved with hands-on activities in which students make a pair of petri dishes or trays; one with dry biocrust, and a second with a loosely packed soil. The students can than test these petri dishes with wind or water forces; using a small fan (or even blowing on the dishes), or simulating rain (e.g., with a watering can). In both cases, the students can observe how biocrusts bind the soil and prevent wind and water erosion. Biocrusts can link these processes to the community of organisms within that petri dish to help students conceptualize how biocrust filaments and plant roots function in similar ways to protect soil from erosion (Figure 2).

### Scale

You cannot always take your classroom out into the world, but you can bring the world into your classroom with biocrusts. The biocrust-filled petri dish, terrarium containing living biocrust communities, or photographs of multiple dynamic species that students can observe are true ecosystems at the small scale, with all major trophic levels and major ecosystem functions (Bowker et al., 2014, 2016; Maestre et al., 2016). This micro-model of a terrestrial ecosystem can be used to introduce systems thinking and build on the objective of developing a deeper understanding of how scale can influence processes on the landscape. Similarly, this micro-ecosystem can be scaled all the way to global processes and global change drivers. This could include discussions of how and why different biocrusts live in different climates (Samolov et al., 2020), how biocrusts interact with the living and abiotic landscapes around them (Rodriguez-Caballero et al., 2019), and how their activities at the small scale could be affecting function at the global scale (Ferrenberg et al., 2017).

## Discussion

In addition to the direct interest from teachers, lesson plans and outreach efforts that use biocrusts can meet broader impact goals of many granting agencies. Education and outreach about biocrusts is at an all-time high with attention across a variety of media sources. Slogans such as “Do not bust the crust” have become a familiar refrain in natural areas where biocrusts are common. Classroom activities linking biocrust with foundational ecological and biological concepts can help instructors develop and students experience an active learning approach, which has been shown to enhance student learning and knowledge retention (Kvam, 2000). When looking at organisms (Figure 2), students can easily identify taxa (e.g., cyanobacteria, mosses, and lichens), learn about the complexities of biodiversity we cannot see, and consider diverse growth forms with differences in morphology and with similarities and differences in their function, and this information can be used to have students dissect what it means to be a species. These questions link directly to evolutionary concepts, and the close physical proximity of organisms in biocrust communities leads naturally to the topic of coexistence of species (Figure 2). Different types of biocrust organisms living together can be used to learn about whether the species are competing with or helping one another as they live side by side (Figure 2). These components can then be related to successional trajectories over time (Figure 2). Students can also actively manipulate the composition of biocrust communities (Figure 2) and, by adding water, can experience the rapid shift in activity of organisms that only moments before were dormant; or they can conduct the bare soil and biocrust fan experiment (as stated earlier) to see how soil is eroded over time learning about patterns and processes (Figure 2). Finally, students can tie these observations to a larger scale while sitting at a computer and looking at how much of Earth’s land area (over 40+%!) is drylands where large fractions can potentially be colonized by biocrusts (Rodriguez-Caballero et al., 2018a). With that much potential to be harnessed in the communities that make up biocrusts as an educational tool, numerous questions can be asked by students, such as “how do different types of land use or different management actions change biocrusts, and how do those changes affect a patch of ground, the ecosystem, or even the world?” These are all concepts teachers can demonstrate in the classroom while providing students learning-by-doing experiences. Using biocrusts as a model system in the classroom can raise the interest in students about biological concepts and inspire individuals to enter into STEM fields and further our knowledge of the natural world.

## Data Availability Statement

The original contributions presented in the study are included in the article/supplementary material, and further inquiries can be directed to the corresponding author.

## Author Contributions

AF conducted the majority of the writing, with all authors providing suggestions and edits, thus enhancing the document. AA, CH, and AF created the figures and AA provided the photos used in the figures unless otherwise noted. All authors contributed to the article and approved of the submitted version.

### Conflict of Interest

The authors declare that the research was conducted in the absence of any commercial or financial relationships that could be construed as a potential conflict of interest.

## Footnotes

^1^https://www.nextgenscience.org/three-dimensions

^2^https://anitaantoninka.wixsite.com/biocrustrestoration
